# Coordinated cell motility is regulated by a combination of LKB1 farnesylation and kinase activity

**DOI:** 10.1038/srep40929

**Published:** 2017-01-19

**Authors:** S. Wilkinson, Y. Hou, J. T. Zoine, J. Saltz, C. Zhang, Z. Chen, L. A. D. Cooper, A. I. Marcus

**Affiliations:** 1 Winship Cancer Institute of Emory University, Department of Hematology and Medical Oncology, Atlanta, GA, USA; 2 Graduate Program in Cancer Biology, Emory University, Atlanta, GA, USA; 3 Department of Biomedical Informatics, Emory University, Atlanta, GA, USA; 4 Department of Biomedical Informatics, Stony Brook University, Stony Brook, NY, USA; 5 Department of Biostatistics and Bioinformatics, Emory University, Atlanta, GA, USA.

## Abstract

Cell motility requires the precise coordination of cell polarization, lamellipodia formation, adhesion, and force generation. LKB1 is a multi-functional serine/threonine kinase that associates with actin at the cellular leading edge of motile cells and suppresses FAK. We sought to understand how LKB1 coordinates these multiple events by systematically dissecting LKB1 protein domain function in combination with live cell imaging and computational approaches. We show that LKB1-actin colocalization is dependent upon LKB1 farnesylation leading to RhoA-ROCK-mediated stress fiber formation, but membrane dynamics is reliant on LKB1 kinase activity. We propose that LKB1 kinase activity controls membrane dynamics through FAK since loss of LKB1 kinase activity results in morphologically defective nascent adhesion sites. In contrast, defective farnesylation mislocalizes nascent adhesion sites, suggesting that LKB1 farnesylation serves as a targeting mechanism for properly localizing adhesion sites during cell motility. Together, we propose a model where coordination of LKB1 farnesylation and kinase activity serve as a multi-step mechanism to coordinate cell motility during migration.

LKB1 (liver kinase B1; also known as STK11) is a multifunctional, serine/threonine kinase that serves as the upstream activator of 14 members of the AMPK (5′ AMP-activated protein kinase) family to regulate energy sensing^1^,^2^, cell motility^3^,^4^, polarity^5^,^6^,^7^, adhesion^5^,^8^,^9^,^10^, and axon differentiation^11^,^12^. In lung adenocarcinoma, LKB1 is the 2nd most-commonly mutated tumor suppressor where the majority of mutations (~72%) are inactivating truncation mutations found within its kinase domain^13^,^14^,^15^,^16^,^17^. Although LKB1 loss is correlated with increased tumor burden and metastasis in a murine model^18^, how LKB1 inactivation impacts its function remains poorly understood.

LKB1 has three major protein domains: the kinase, N-terminal (NTD), and C-terminal (CTD) domains. The LKB1 kinase domain is responsible for phosphorylating and activating the AMPK family, while the LKB1 CTD contains multiple phosphorylation residues and a C-terminal farnesylation motif (amino acids 430–433 in human, 433–436 in murine model) for post-translational membrane targeting^19^,^20^. LKB1 phosphorylation at residue S431 in murine models (90% homology to human^21^) does not affect its farnesylation, suggesting that farnesylation is functionally distinct from phosphorylation^22^. Although LKB1 kinase activity is not impacted by farnesylation^22^, *in vivo* studies suggest farnesylation promotes membrane localization to activate myristoylated AMPK, highlighting the role of post-translational farnesylation in localizing LKB1 kinase activity^20^.

Several studies have implicated LKB1 as a major regulator of cell polarity and downstream motility. Restoring LKB1 activity in single epithelial cells induces cellular polarization with an acinar actin cap even in the absence of cell:cell contacts^23^. Cell biological studies show that upon activation in lung cancer cells, LKB1 rapidly translocates to the cellular leading edge, where it associates with actin in lamellipodia^24^. LKB1 promotes stress fiber assembly in contractile cells to help drive actin dynamics during cell motility^25^. These events are likely through small Rho-GTPases^24^,^26^, where LKB1 signals to RhoA to drive mesenchymal polarization during 3D invasion in a farnesylation-dependent but kinase-independent manner^5^. Although LKB1 colocalizes with actin at the leading edge and regulates Rho-GTPase activity to drive polarity, the functional significance of these events in the context of cell motility remains largely unstudied.

Recent *in vivo* and *in vitro* experiments show LKB1 loss also leads to adhesion defects, specifically FAK hyperphosphorylation^5^,^8^,^9^,^10^,^18^,^27^. LKB1 depletion results in individual FAK sites that fail to mature properly^9^,^10^ and is overseen through an LKB1-MARK1/4-FAK pathway^9^. Further, recent studies highlight the role of FAK in promoting lamellipodia protrusion through nascent adhesion (NA) assembly^28^. Together, these highlight the major question of how LKB1 coordinates its actin-based function described above with its role in cell adhesion during motility; therefore, the goal of these studies was to examine how the different LKB1 protein domains impact the interplay between its role on actin and focal adhesion function during cell motility. Our data support a model whereby LKB1 farnesylation, independent of its kinase activity, promotes its cytoplasmic actin co-localization and retrograde actin flow through a RhoA-Rho-associated protein kinase (ROCK) pathway to induce actin stress fiber assembly. In contrast, LKB1 kinase activity regulates membrane dynamics and represses membrane ruffling. When we examine LKB1 within nascent lamellipodia, we show that LKB1 farnesylation localizes LKB1 to the membrane, where LKB1 kinase activity then regulates NA formation and deposition. Together, we propose a model where coordination of LKB1 farnesylation and kinase activity serve as a multi-step mechanism to coordinate cell motility during migration.

## Results

### LKB1 farnesylation is required to promote actin stress fiber formation through RhoA signaling

**Figure 1:**
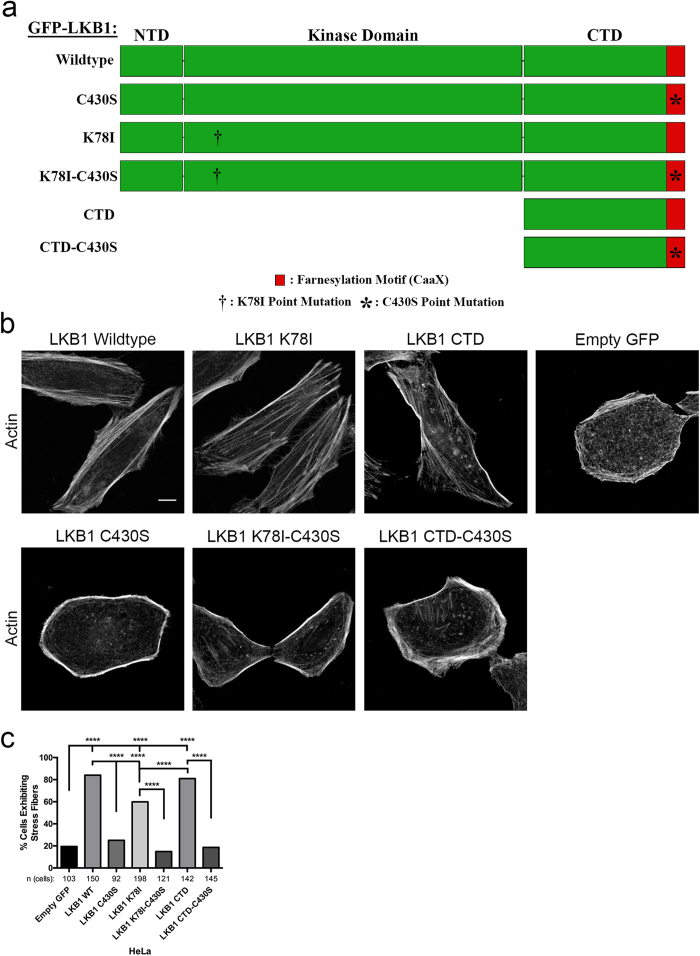
LKB1 promotes actin stress fibers in a farnesylation-dependent manner. (**a**) Schematic showing HeLa cells that were transfected to express GFP-tagged: wildtype LKB1, a C430S mutation to disrupt farnesylation, a K78I kinase dead mutation, a double mutation with both K78I and C430S, the C-terminal domain (CTD) alone, or the CTD alone with a C430S mutation. (**b**) Immunofluorescent images of HeLa cells transfected with GFP-LKB1 and stained with phalloidin-555. (**c**) The percentage of cells containing lateral stress fibers was quantified 24 hours after transfection. Significance was measured between comparisons using a 2-tailed Chi-squared analysis with a p-value of 0.05, where ****p ≤ 0.0001. Scale bar: 10 μm.

To probe how different LKB1 domains impact actin stress fiber formation, we created a series of LKB1 mutants that modify LKB1 farnesylation and kinase activity. GFP-tagged: wildtype LKB1, a C430S mutation to disrupt farnesylation, a K78I kinase-dead mutation^29^, the CTD alone (kinase dead as well), and the CTD with a C430S mutation (Fig. 1a, ref. 5) were transiently re-expressed in HeLa (LKB1-null) cells. Empty GFP control cells exhibit a predominantly amoeboid phenotype with only 19.4% of cells exhibiting lateral stress fibers spanning the cell length (Fig. 1b,c, Supplementary Figure 1); however, upon re-expression of wildtype LKB1, cells revert to a mesenchymal morphology with 84% of cells exhibiting lateral stress fibers (Fig. 1b,c, Supplementary Figure 1). Preventing LKB1 farnesylation with a C430S mutation resulted in reduced stress fiber assembly compared to LKB1 wildtype cells, with only 25% of cells exhibiting lateral stress fibers (Fig. 1b,c, Supplementary Figure 1). Disrupting kinase activity with the LKB1 K78I mutant induced less stress fiber assembly (59.9%) than LKB1 wildtype, though the lack of stress fiber assembly was more apparent when disrupting farnesylation alone (LKB1 C430S, 25%) or disrupting both farnesylation and kinase activity in the double mutant (LKB1 K78I-C430S, 14.9%). Expression of the LKB1 CTD alone promoted stress fiber assembly (80.9%), but this effect was abolished (18.6%) with a single point mutation in the CTD farnesylation site (CTD-C430S; Fig. 1b,c, Supplementary Figure 1). Together, these data indicate that the LKB1 CTD alone can induce stress fiber assembly and LKB1 farnesylation is critical for this to occur.

**Figure 2:**
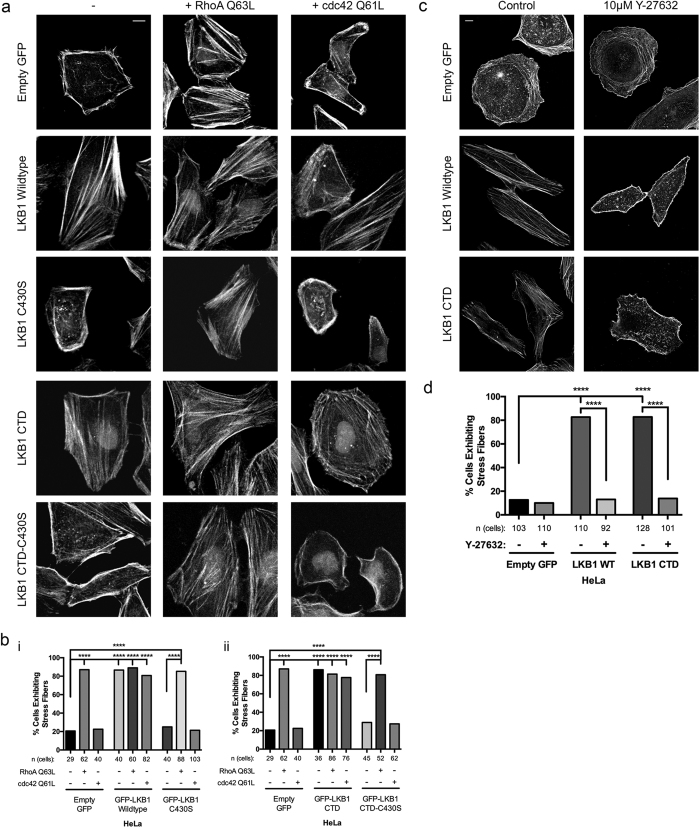
LKB1 signals via the RhoA-ROCK pathway to promote stress fiber assembly. (**a**) Immunofluorescent images of HeLa cells expressing GFP-LKB1 alone (−) or while co-expressing constitutively active RhoA (Q63L) or constitutively active cdc42 (Q61L) and stained with phalloidin-555. (**b**) Percent of cells containing lateral stress fibers was quantified 24 hours after transfection. (i) Percentage of cells containing stress fibers for empty GFP control, GFP-LKB1 Wildtype, and GFP-LKB1 C430S with their respective Rho-GTPase mutants. (ii) Percentage of cells containing stress fibers for empty GFP control, GFP-LKB1 CTD, and GFP-LKB1 CTD-C430S with their respective Rho-GTPase mutants. (**c**) Immunofluorescent images of HeLa cells expressing GFP-LKB1 with or without 10 μM Y-27632 ROCK inhibitor treatment and stained with phalloidin-555. (**d**) Percentage of cells containing stress fibers in response to ROCK inhibitor treatment. Significance was measured between comparisons using a 2-tailed Chi-squared analysis with a p-value of 0.05, where ****p ≤ 0.0001. Scale bar: 10 μm.

LKB1 signals to RhoA, and RhoA is required for stress fiber assembly through ROCK and subsequent myosin light chain phosphorylation^25^,^26^,^30^,^31^; therefore, we sought to determine whether LKB1 farnesylation promotes stress fiber assembly through RhoA. Using HeLa cells stably expressing constitutively active RhoA or cdc42 and co-expressing members of the panel of LKB1 plasmids (Fig. 1a), we observe activating either RhoA or cdc42 in cells re-expressing wildtype LKB1 or the LKB1 CTD did not impact mesenchymal morphology and lateral stress fiber formation with cells maintaining this LKB1-induced phenotype (Fig. 2a,b, Supplementary Figure 1). However, restoring RhoA activity in either empty GFP control or farnesyl-mutant cells induced a rescue of lateral stress fiber assembly, suggesting LKB1 farnesylation lies upstream of RhoA activity in the stress fiber assembly pathway (Fig. 2a,b, Supplementary Figure 1). Furthermore, restoring cdc42 activity in empty GFP or farnesyl-compromised cells failed to rescue the stress fiber phenotype, highlighting the role of LKB1 farnesylation in signaling to RhoA and not cdc42 to drive stress fiber assembly.

To determine how LKB1 drives stress fiber assembly through RhoA, a similar experiment was performed in the presence of the ROCK inhibitor Y-27632. As expected, ROCK inhibition had limited impact on empty GFP control cells with 10% (vs 12.6% untreated control) of cells exhibiting lateral stress fibers upon ROCK inhibition. However, in cells re-expressing both wildtype LKB1 and the LKB1 CTD alone, ROCK inhibition abrogated the stress fiber assembly rescue by reducing the percent of cells containing stress fibers from 82.6% to 13.6% in LKB1 WT and 82.2% to 13.5% in LKB1 CTD (Fig. 2c,d, Supplementary Figure 1). Together, these data suggest LKB1 farnesylation promotes lateral stress fiber assembly through a RhoA-ROCK signaling pathway.

### LKB1 colocalizes with actin at the cell periphery through C-terminal domain farnesylation

LKB1 associates with actin at the cell periphery of motile cells^24^; however, how this is regulated and the functional consequences of the LKB1-actin association have not been investigated. Using the LKB1 panel described in Fig. 1a, we noted no significant difference in cell footprint based on LKB1 status (Supplementary Figure 2). However, while examining cell area, we noticed striking differences in LKB1 subcellular localization; therefore, using quantitative imaging we generated a cytoplasmic:nuclear ratio (C:N ratio) of LKB1 localization, where a C:N ratio of 1 indicates equal GFP:LKB1 signal in the nucleus and cytoplasm, while a C:N higher than 1 indicates greater cytoplasmic GFP:LKB1 localization. When re-expressing wildtype LKB1, a C:N ratio of 0.51 was observed, indicating that the basal LKB1 signal was two times higher in the nucleus than the cytoplasm. However, when disrupting LKB1 farnesylation with a C430S mutation (or K78I-C430S or CTD-C430S), the signal shifted more into the nucleus with a C:N ratio of 0.25 (Supplementary Fig. 2), suggesting that LKB1 farnesylation promotes its cytoplasmic localization. The K78I LKB1 mutant or the LKB1 CTD-only truncate had no impact on GFP:LKB1 localization, suggesting that kinase domain functionality does not impact LKB1 nuclear or cytoplasmic localization.

**Figure 3:**
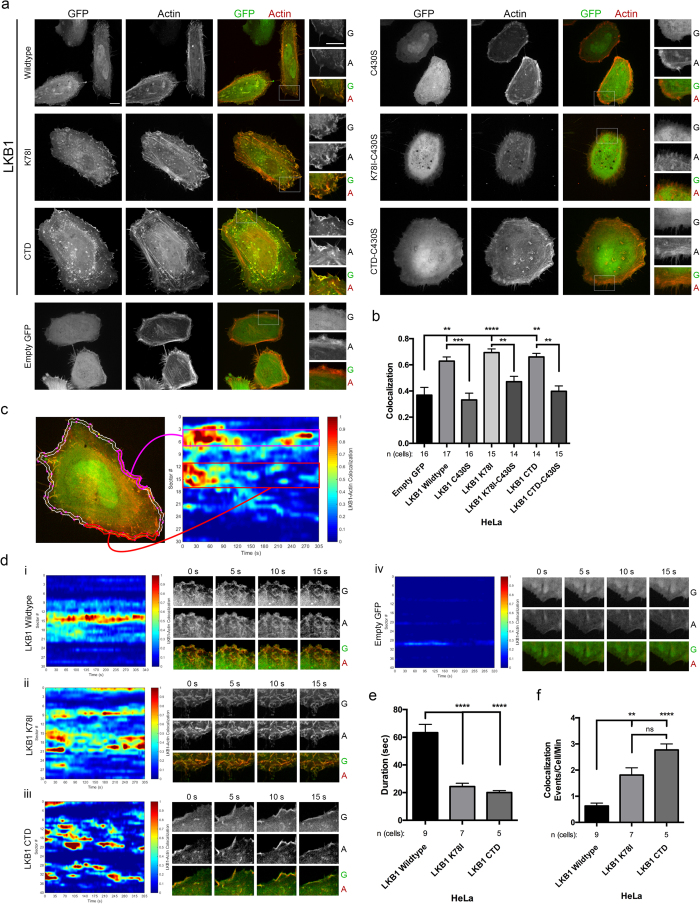
LKB1 farnesylation and kinase activity combine to drive actin colocalization over time. (**a**) Live-cell images of HeLa cells transfected with GFP-LKB1 and LifeAct-mRuby. Inserts are cropped from the white box in the merged image. G: GFP; A: Actin. (**b**) LKB1-actin colocalization values for the respective constructs from (**a**). (**c**) Cellular Analysis of Dynamic Events (CADE) was used (see methods). Briefly, live-cell movies were segmented to identify the cell border and a region 2 μm interior of that border. Spatiotemporal colocalization was then measured for the duration of the image acquisition and plotted. Different sectors of the cell boundary are on the y-axis, and time is on the x-axis. Red indicates strong colocalization, blue indicates no colocalization. (**d**) (i–iii) CADE analysis of various LKB1 constructs. (iv) CADE analysis of empty GFP control. (**e**) Graph of the average duration of colocalization events/condition. (**f**) Graph of the average number of colocalization events/cell/minute for each condition. A Kruskall-Wallis test with a p-value of 0.05 showed statistical significance. Significance was then measured between comparisons using the Dunn’s multiple comparisons test with a p-value of 0.05, where **p ≤ 0.01; ***p ≤ 0.001; ****p ≤ 0.0001. Scale bar: 10 μm. Error bars = SEM.

Using live-cell imaging, we confirmed that cytoplasmic LKB1 colocalizes with actin in living HeLa cells primarily in the cell periphery with smaller actin filaments (Fig. 3a,b). This colocalization originates at the membrane and travels towards the cell interior, presumably through actin retrograde flow. Since LKB1-actin colocalization occurs proximal to the cell membrane with primarily cortical actin filaments, we wanted to determine if LKB1 farnesylation mediates its actin colocalization. When LKB1 farnesylation is disrupted with the C430S mutant, we observed that the LKB1-actin colocalization is almost completely abrogated, returning to empty GFP control levels (Fig. 3a,b). The impact of LKB1 kinase activity on LKB1-actin colocalization was then tested by re-expressing a K78I kinase-dead mutant. In this case, similar LKB1-actin colocalization as wildtype LKB1 was observed, suggesting that kinase activity is not required for its actin association (Fig. 3a,b).

We next wanted to determine if the LKB1 CTD alone, which contains the farnesylation motif but lacks kinase activity, is responsible for its actin colocalization. Re-expression of the LKB1 CTD alone led to extensive LKB1-actin colocalization, again primarily with short actin filaments traveling towards the cell interior (Fig. 3a,b). Importantly, the LKB1 CTD-C430S farnesylation mutant completely abolished the LKB1-actin colocalization (Fig. 3a,b), further supporting a critical role for farnesylation in promoting LKB1-actin colocalization.

Given that the LKB1-actin association appears to originate at the membrane and maintains this colocalization while traveling towards the cell interior, we assessed this association over time using live cell confocal imaging of the GFP-tagged LKB1 constructs (Fig. 1a) and LifeAct-mRuby, using Cellular Analysis of Dynamic Events (Fig. 3c, CADE, see methods). When examining LKB1 colocalization over time, re-expression of wildtype LKB1 induced a persistent actin colocalization, with the LKB1-actin colocalization event averaging 63 s (Fig. 3d(i),e, Supplementary Fig. S3). A similar analysis was done with the LKB1 K78I kinase dead mutant and the LKB1 CTD (lacking the kinase domain). As shown previously, inhibition of LKB1 kinase activity did not impact its actin colocalization; however, it does impact actin colocalization persistence. Specifically, when re-expressing the LKB1 K78I kinase dead mutant or the CTD alone (also kinase dead), the LKB1-actin colocalization pattern shifts from persistent dynamics averaging over 60 s to transient associations lasting 24.3 s and 20.0 s, respectively (Fig. 3d(ii,iii), Supplementary Fig. S3). In addition, the persistent colocalization with wildtype LKB1 results in only 0.6 colocalization events/cell/minute, while disrupting kinase activity increases colocalization events to 1.8 (LKB1 K78I) and 2.8 (LKB1 CTD) events/cell/minute (Fig. 3f). These results suggest that abolishing farnesylation results in loss of actin colocalization while loss of kinase activity reduces actin colocalization persistence.

### LKB1 kinase activity represses membrane dynamics and traveling membrane waves through FAK

**Figure 4:**
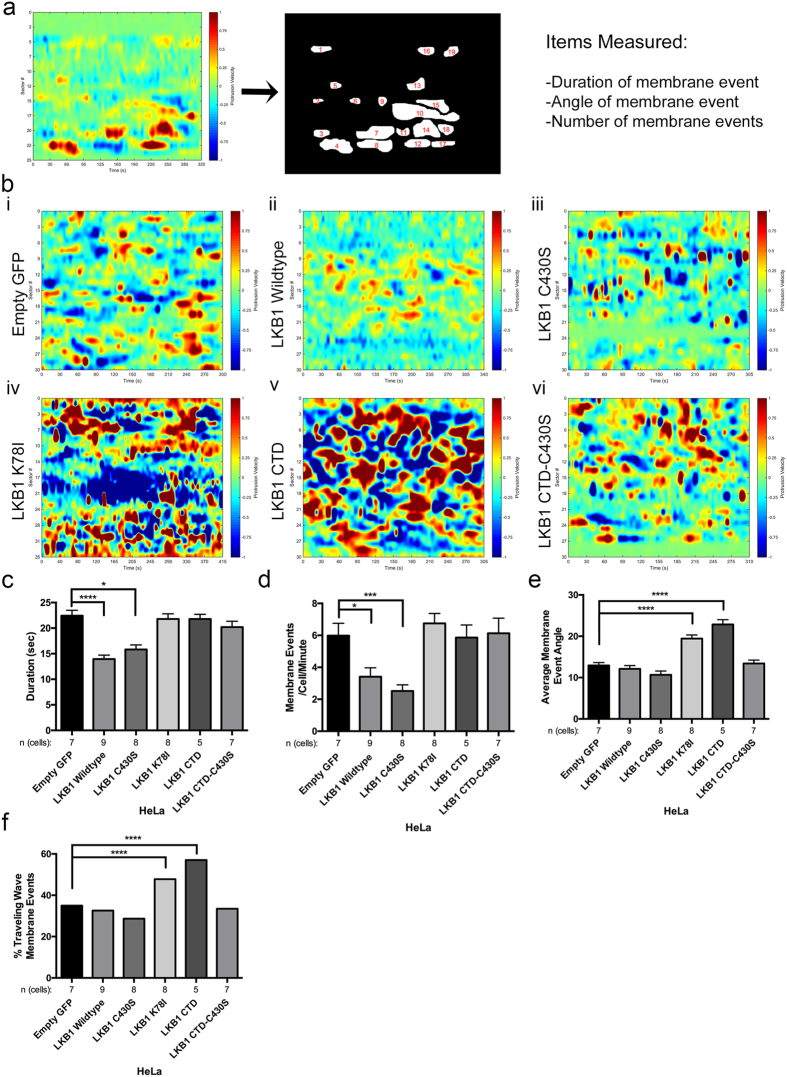
LKB1 kinase activity regulates cell membrane dynamics. (**a**) Membrane protrusion (red) and retraction (blue) events were measured around the cell periphery using the same CADE analysis as in Fig. 3c. Cell boundary sectors are on y-axis, time is on x-axis. Protrusion and retraction events were manually identified and the duration, angle, and number of membrane events/cell/minute was quantified. (**b**) (i) CADE analysis on empty GFP control cells. (ii–vi) CADE analysis on LKB1 and the various LKB1 mutants. (**c**) Duration of membrane events (seconds). (**d**) Average number of membrane events/cell/minute. (**e**) Average membrane event angle. (**f)** Percent of membrane events exhibiting traveling waves, defined as an angle greater than 10°. In parts (**c**–**e**), a Kruskall-Wallis test with a p-value of 0.05 showed statistical significance. Significance was then measured between comparisons using Dunn’s multiple comparisons test with a p-value of 0.05. In part (**f**), significance was measured using a 2-tailed Chi-squared analysis with a p-value of 0.05. *p ≤ 0.05; ***p ≤ 0.001; ****p ≤ 0.0001. Error bars = SEM.

Since actin assembly is critical for lamellipodia elongation^32^, we determined how the LKB1-actin colocalization impacts membrane protrusion and retraction events by observing membrane dynamics with the LKB1 truncates and mutants. Using CADE analysis, empty GFP control cells exhibit an average membrane event (defined as either a membrane protrusion or retraction) for a duration of 22 ± 1.1 seconds (Fig. 4a, b(i), Supplementary Fig. S4); however, upon re-expression of wildtype LKB1 or a C430S farnesyl mutant, cells had significantly reduced mean membrane event duration to 14 ± 0.8 s and 16 ± 0.9 s, respectively, (Fig. 4b(ii–iii), Supplementary Fig. S4). Re-expressing wildtype LKB1 or the C430S mutant also resulted in a decreased number of membrane events per cell, from 6.0 ± 0.8 events/cell/minute for empty GFP to 3.4 ± 0.6 events/cell/min and 2.5 ± 0.4 events/cell/min, respectively (Fig. 4d). Although farnesylation does not impact membrane dynamics, disrupting LKB1 kinase activity with either a K78I mutation or the LKB1 CTD alone (also lacking kinase activity), induced a reversion back to membrane event durations and number of events/cell/minute similar to empty GFP control (Fig. 4c,d, Supplementary Fig. S4). Together, these data indicate the LKB1 kinase activity, independent of its farnesylation, is responsible for repressing both duration of membrane activity and number of membrane events/cell.

Traveling membrane waves are defined as a cell membrane protrusion traveling laterally along the membrane, and are typically associated with leading edge actin polymerization^33^,^34^,^35^,^36^. We examined traveling wave formation during protrusion/retraction with our panel of LKB1 constructs (Fig. 1a). To do this, we quantified the angle of membrane events by defining a traveling wave as any membrane event with an angle greater than 10°. In empty GFP control cells, protrusion/retraction events generally occur within the same region of the cell, with only 35.0% of all membrane events exhibiting traveling waves (mean event angle of 12.9 ± 0.7°) (Fig. 4e,f). Similarly, cells re-expressing wildtype LKB1 and the LKB1 C430S mutant exhibited 32.6% and 28.7% of membrane events as traveling waves, with an average membrane event angle of 12.1 ± 0.8° and 10.7 ± 0.9°, respectively (Fig. 4e,f). In contrast, re-expressing either the LKB1 K78I (kinase dead) or the LKB1 CTD alone (lacking the kinase domain) resulted in greater traveling waves with 47.8% and 57.0% of membrane events exhibiting traveling waves, with an average angle of 19.5 ± 0.9° and 22.9 ± 1.2°, respectively (Fig. 4e,f). The CTD-C430S construct also resulted in abrogation of traveling waves, potentially due to the lack of farnesylation to target LKB1 to the membrane. Together, these data suggest that LKB1 functions in a farnesylation-independent, but kinase-dependent manner to repress traveling membrane waves during protrusion and retraction events.

**Figure 5:**
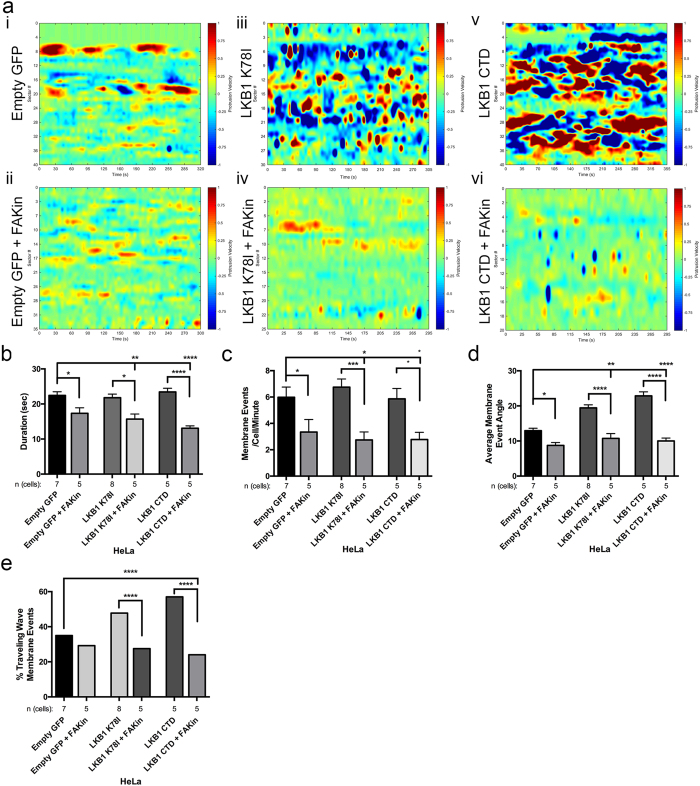
LKB1 signals to FAK to regulate cell membrane dynamics. (**a**) Cells were treated with 1 μM PF-562271 FAK inhibitor and imaged to examine membrane dynamics. (i) CADE analysis on untreated empty GFP control cells exhibit basal level membrane events. (ii) Analysis of empty GFP control with FAK inhibitor. (iii) Untreated kinase dead LKB1 K78I. (iv) LKB1 K78I with FAK inhibitor. (v) Untreated LKB1 C-terminal domain. vi) LKB1 CTD with FAK inhibitor. (**b**) Duration of membrane events (seconds). (**c**) Average number of membrane events/cell/minute. (**d**) Average membrane event angle. (**e**) Percent of membrane events exhibiting traveling waves, defined as an angle greater than 10°. In parts (**b**–**d**), a Kruskall-Wallis test with a p-value of 0.05 showed statistical significance. Significance was then measured between comparisons using Dunn’s multiple comparisons test with a p-value of 0.05. In part (**e**), significance was measured using a 2-tailed Chi-squared analysis with a p-value of 0.05. *p ≤ 0.05; **p ≤ 0.01; ***p ≤ 0.001; ****p ≤ 0.0001. Error bars = SEM.

Since LKB1 kinase activity represses membrane dynamics (Fig. 4) and pFAK^5^,^9^,^10^, we determined whether LKB1 represses membrane dynamics through FAK activity. To do this, cells were treated with the pharmacological FAK inhibitor PF-562271^37^. Empty GFP control cells treated with 1 μM FAK inhibitor show a reduced duration of membrane events from 22.4 ± 1.1 s to 17.3 ± 1.6 s and a reduced number of events/cell from 30.6 ± 0.8 to 17.3 ± 1.0 events/cell (Fig. 5a(i–ii), Supplementary Fig. S5). In cells re-expressing the LKB1 K78I kinase dead mutant or the LKB1 CTD alone (also lacking kinase activity), FAK inhibition also reduced the duration of membrane events, from 21.8 ± 1.0 s to 15.7 ± 1.5 s (LKB1 K78I) and from 23.4 ± 1.1 s to 13.1 ± 0.7 s (LKB1 CTD), as well as reducing the number of membrane events/cell from 6.8 ± 0.6 to 2.8 ± 0.6 events/cell/minute (LKB1 K78I) and 5.9 ± 0.8 to 3.2 ± 0.9 events/cell/minute (LKB1 CTD) (Fig. 5a(iii–vi),b,c, Supplementary Fig. S5). Together, these data show that FAK inhibition significantly decreased membrane activity in kinase activity-compromised cells, suggesting that the FAK pathway is at least in part responsible for driving membrane activity in these cells.

To probe the mechanism underlying traveling waves, we examined the angle of the traveling membrane waves in response to FAK inhibition. Although 35.0% membrane events in empty GFP control cells exhibited traveling waves, FAK inhibition reduced the percentage to 29.2%, with reduction in the average angle of these waves from 12.9 ± 0.7° to 8.7 ± 0.8° (Fig. 5d,e). In addition, when inhibiting LKB1 kinase activity, the percent of traveling waves decreased from 47.8% to 27.5% (K78I) and from 57.0% to 24.0% (CTD), with the average angle of the waves also reduced from 19.5 ± 1.0° to 10.7 ± 1.5° (LKB1 K78I), and 22.9 ± 1.1° to 10.0 ± 0.7° (LKB1 CTD) (Fig. 5d,e). Together, these data indicate membrane activity is impacted by FAK activity, as disrupting FAK inhibits membrane events and traveling waves associated with these events.

### LKB1 farnesylation localizes kinase activity to the leading edge membrane to promote nascent adhesion site assembly and maturation

**Figure 6:**
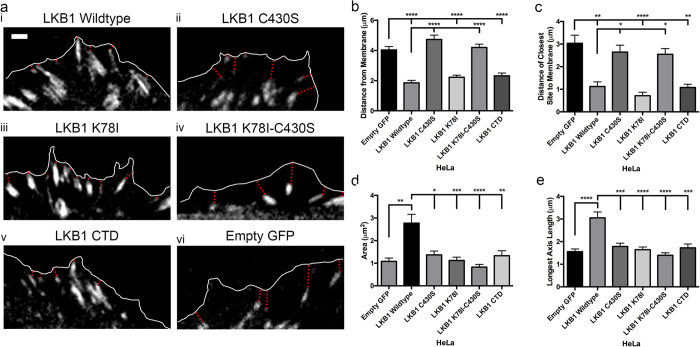
A combination of LKB1 farnesylation and kinase activity spatially regulates adhesion site development in emerging lamellipodia. (**a**) Live-cell imaging of mRuby-Paxillin within emerging lamellipodia identify nascent adhesion (NA) site development. White line indicates cell boundary; red dotted line indicates distance from adhesion site to membrane. (i–v) Localization and size of paxillin sites in the various LKB1 constructs. (vi) Localization and size of paxillin sites in empty GFP control. (**b**) Average NA site distance (μm) from the membrane. (**c**) Distance (μm) of the nearest NA site to the leading edge membrane. (**d**) Average NA site area (μm^2^). (**e**) Average length (μm) of the longest axis in the NA sites. A Kruskall-Wallis test with a p-value of 0.05 showed statistical significance. Significance was then measured between comparisons using Dunn’s multiple comparisons test with a p-value of 0.05, where *p ≤ 0.05; **p ≤ 0.01; ***p ≤ 0.001; ****p ≤ 0.0001. N = 10 cells/condition. Scale bar: 2 μm. Error bars = SEM.

Our data suggests that LKB1 regulates membrane dynamics through FAK (Figs 4 and 5). Since LKB1 farnesylation targets LKB1 to the membrane, and nascent adhesion (NA) sites form at or near the membrane of newly formed lamellipodia, we tested the hypothesis that LKB1 farnesylation serves as a targeting mechanism for placing NA sites at or near the membrane. Using mRuby-Paxillin to monitor adhesion site localization over time, we observed that empty GFP control cells contained NA sites throughout the lamellipodia, with an average NA site distance to the leading edge membrane of 4.0 μm and the closest site 3.0 μm from the membrane (Fig. 6a–c). However, re-expressing wildtype LKB1 localizes the NA sites significantly closer to the leading edge of the membrane, with an average distance of 1.9 μm and the closest site 1.2 μm from the leading edge (Fig. 6a(i),b,c). Disrupting kinase activity with the K78I mutant or CTD truncate did not impact NA site localization; however, when disrupting farnesylation with either the C430S or K78I-C430S mutations, the average distance to the membrane reverted back to 4.7 μm and 4.2 μm from the membrane and the closest NA sites to the membrane at 2.7 μm and 2.5 μm, respectively (Fig. 6a–c). Together, these data suggest that LKB1 farnesylation is required for targeting of NA sites at or near the membrane of a newly-formed lamellipodia.

NA site morphology was also quantified and we observed an increase in NA site area from 1.1 μm^2^ with empty GFP control to 2.8 μm^2^ with re-expressed wildtype LKB1 (Fig. 6d). Additionally, re-expression of wildtype LKB1 promoted elongated NA sites of 3.1 μm in length, compared to 1.6 μm in empty GFP control (Fig. 6e). We next probed how LKB1 kinase activity impacts NA site maturation, whereby both the K78I kinase inactive mutant and the CTD alone (lacking kinase activity) abrogated the increase in NA site area and elongation observed with wildtype LKB1 (Fig. 6d,e). Interestingly, disrupting farnesylation with the C430S mutant also abrogated NA maturation, with NA site trends similar to empty GFP control and kinase dead mutants (Fig. 6d,e). Together, these data indicate that LKB1 kinase activity and farnesylation are required to promote NA site maturation during cell motility.

### Coordination of LKB1 farnesylation and kinase activity to direct cell motility

**Figure 7:**
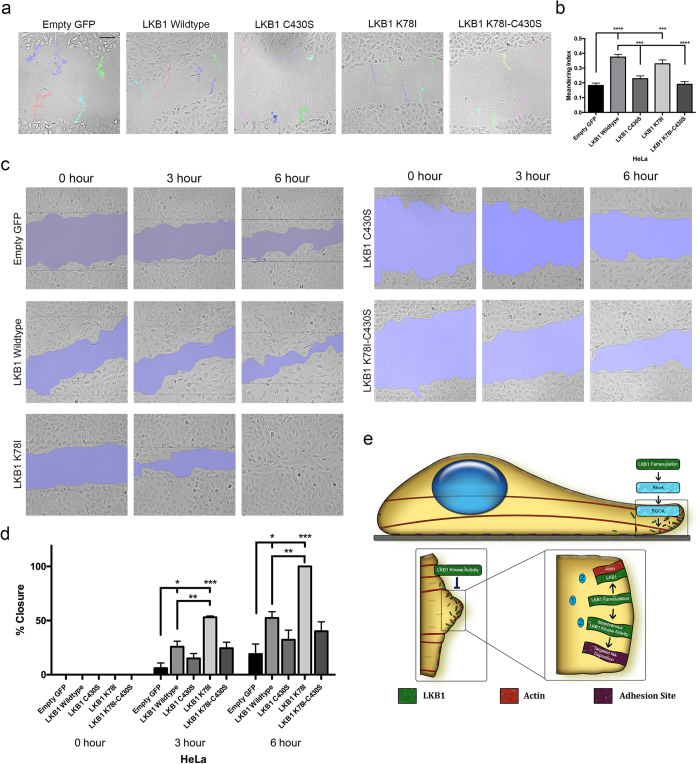
LKB1 farnesylation and kinase activity coordinate cell motility. (**a**) Live-cell imaging of scratch wound assays using HeLa cells stably expressing our panel of GFP-LKB1 plasmids. Representative meandering indices shown for each construct. (**b**) The meandering index for each cell line was calculated and shown as a bar graph. (**c**) Area closed for each cell line at 0, 3, and 6 hours post-scratch. (**d**) The percentage of area closed at 0, 3, and 6 hours was calculated and shown as a bar graph. (**e**) Model: LKB1 farnesylation promotes its leading edge actin colocalization and signals via RhoA-ROCK to promote stress fiber formation, while LKB1 kinase activity regulates membrane dynamics. Together, LKB1 farnesylation localizes LKB1 to leading edge lamellipodia where LKB1 kinase activity can then regulate nascent adhesion formation and deposition. In part (**b**), a Kruskall-Wallis test with a p-value of 0.05 showed statistical significance. Significance was then measured between comparisons using Dunn’s multiple comparisons test with a p-value of 0.05. In part (**d**), wound closure passed the assumptions of parametric tests (homogeneity of variance) and was thus measured using ANOVA followed by Sidak’s multiple comparisons test with a p-value of 0.05. *p ≤ 0.05; **p ≤ 0.01; ***p ≤ 0.001; ****p ≤ 0.0001. N = 30 cells/condition for meandering; N = 3 scratches/condition for closure. Scale bar: 100 μm. Error bars = SEM.

We next determined the impact of LKB1 status on cell motility. Using the panel of HeLa cells stably re-expressing LKB1 (Fig. 1a), scratch wound assays were performed to monitor cell motility over time. When re-expressing wildtype LKB1 compared to empty GFP control, cells exhibited a higher meandering index during migration, suggesting wildtype LKB1 promotes directed migration during 2D motility (Fig. 7a,b). This is consistent with previous data examining LKB1 function during 3D invasion^5^. Similarly, re-expressing a kinase-dead (K78I) LKB1 or the LKB1 CTD alone (also kinase dead) rescued the meandering index compared to empty GFP control, indicating that directed cell motility occurs independent of LKB1 kinase activity (Fig. 7a,b). When LKB1 farnesylation is abrogated with the C430S, K78I-C430S, or CTD-C430S mutants, cells have a lower meandering index (Fig. 7a,b). Together, these data indicate that LKB1 promotes directional persistence in a farnesylation-dependent and kinase-independent manner.

We then determined how LKB1 impacts the rate of wound closure and observe that GFP:LKB1 promotes wound closure over time compared to empty GFP control at both 3 and 6 hours (Fig. 7c,d). When farnesylation is abrogated with either a C430S mutation or the K78I-C430S double mutant, wound closure reverted back to empty GFP control levels (Fig. 7c,d). Disrupting LKB1 kinase activity with a K78I mutation increased wound closure compared to both empty GFP control and wildtype LKB1, with 53% and 100% closure at 3 and 6 hours, respectively (Fig. 7c,d). Together, these data show LKB1 farnesylation is required for directed migration, while kinase activity regulates migration rate.

## Discussion

LKB1 is an actin-associated protein^24^,^25^ that primarily colocalizes with polymerizing cortical actin filaments adjacent to the cell membrane (Fig. 3). After polymerization, actin-associated LKB1 is rapidly translocated towards the cell interior presumably through retrograde flow^38^,^39^. This actin association is farnesylation-dependent, since disrupting LKB1 farnesylation abolishes actin colocalization (Fig. 3) resulting in the loss of stress fibers (Fig. 1), which suggests that LKB1 farnesylation is functionally linked to downstream actin stress fiber formation. Our data indicate that these events are at least in part due to RhoA-ROCK signaling, since a constitutively active RhoA rescues LKB1-farnesylation defects by restoring actin stress fibers (Fig. 2). Additionally, inhibition of the downstream RhoA kinase, ROCK^40^, abolished LKB1-induced stress fiber formation (Fig. 2). This appears consistent with previous RhoA-ROCK pathways^24^,^25^,^26^, placing LKB1 farnesylation and its actin colocalization upstream of canonical RhoA signaling pathways.

In contrast, LKB1 kinase activity is not required for the LKB1-actin association, since disrupting its kinase activity with a K78I mutation or completely removing the LKB1 kinase domain, does not impact actin colocalization. Though surprisingly, LKB1 kinase activity is important for the duration of the LKB1-actin association; specifically, in cells expressing a K78I point mutation in the kinase domain or removal of the kinase domain entirely (CTD alone), LKB1 still colocalizes with actin but these events are more frequent with significantly decreased durations compared to wildtype LKB1 (Fig. 2). These data suggest that while its actin association *per se* is not kinase-dependent, its ability to maintain this colocalization over time is dependent on its kinase activity.

LKB1 serves as the upstream kinase for the AMPK family, which includes MARK1 and MARK4, and phosphorylates MARK1/4 to restrict FAK activity^9^. When LKB1 function is lost, pFAK-Y^397^ becomes hyperphosphorylated, resulting in cells with a higher adhesion that are able to remodel collagen more effectively in a 3D environment^5^,^10^,^41^. We observe in living motile cells that disrupting LKB1 kinase activity with either the K78I kinase mutant or the CTD alone resulted in increased membrane protrusion and retraction events, as well as an increase in membrane traveling waves, that lasted for a greater duration than cells re-expressing wildtype LKB1 (Fig. 4). This would be consistent with the increased adhesion observed in LKB1-compromised cells^5^,^9^,^10^ and supports a more stable and persistent lamellipodia due to regulated adhesion signaling. Additionally, pharmacologic inhibition of FAK signaling relieves these cells of dysregulated membrane activity (Fig. 5), which supports the model that LKB1 kinase activity regulates lamellipodia adhesion through FAK. Specifically, disrupting LKB1 farnesylation significantly reduces NA area and elongation with sites mislocalized further from the membrane of lamellipodia as compared to wildtype LKB1. Interestingly, disrupting kinase activity also results in reduced NA area and elongation, although the immature NA sites remain proximal to the lamellipodial membrane.

Taken together, we propose a model (Fig. 7e, Table 1) whereby LKB1 kinase activity and farnesylation cooperate to regulate actin filament assembly, membrane activity, adhesion dynamics, and subsequent cell migration. First, LKB1 farnesylation allows LKB1 to localize to the membrane, where it colocalizes with actin and signals to the RhoA-ROCK pathway to promote downstream stress fiber assembly. This in turn leads to more directed migration, which is supported by our wound closure data (Fig. 7). Second, LKB1 kinase activity regulates FAK activity, resulting in repression of membrane dynamics and maturation of NA sites. Disrupting kinase activity also promotes increased wound closure rate suggesting that kinase activity alone is important for cell motility velocity, and we speculate could be related to the NA site defects. We hypothesize that this multi-step process begins with LKB1 farnesylation localizing it to the membrane, where its kinase activity then regulates FAK and subsequent NA formation and maturation. When this kinase activity is disrupted, increased adhesion and membrane dynamics ensue. However, our data suggests disrupting LKB1 farnesylation mislocalizes LKB1 kinase activity away from the membrane, thus impacting the ability of LKB1 kinase activity to regulate FAK and NA assembly. These data could be considered analogous to a previous report showing LKB1 farnesylation is critical for promoting AMPK phosphorylation by localizing LKB1 to the membrane for subsequent phosphorylation of AMPK^20^. The well-defined link between FAK and actin^28^,^42^,^43^,^44^ would suggest that the LKB1-actin function may be related to the LKB1-FAK function, given that its farnesylation appears to lie upstream of LKB1 kinase activity to regulate focal adhesion dynamics during cell motility. Together, these data suggest LKB1 farnesylation and kinase activity cooperatively function to promote cell motility.

## Methods

### Cell culture and generating stable cells

HeLa cells (ATCC, Manassas, VA) were cultured in Dulbecco’s Modified Eagle Medium (DMEM) media supplemented with 10% fetal bovine serum and 100 units/mL of penicillin/streptomycin, and maintained at 37 °C and 5% CO_2_.

To generate HeLa cells stably expressing GFP-tagged LKB1 and constitutively active RhoA or cdc42, wildtype LKB1 and the various LKB1 domains and mutations were cloned into a pEGFP-C1 vector. Empty GFP or the GFP-LKB1 constructs were then subcloned from the pEGFP-C1 vector into a pBabe-puro vector. Constitutively active RhoA (Q63L) and cdc42 (Q61L) were subcloned from a pCDNA3 vector into pBabe-Hygro. The pBabe constructs were then transfected into Phoenix-ampho cells with Lipofectamine 2000 and PLUS reagent (Invitrogen, Grand Island, NY). Cells expressing only empty GFP or GFP-LKB1 were selected with 1 μg/ml puromycin, while cells co-expressing the constitutively active RhoA or cdc42 mutants were selected with 1 μg/ml puromycin and 300 μg/ml hygromycin (EMD Millipore, Billerica, MA). Proper expression of GFP-LKB1 was verified using IF and Western blot to confirm phenotype and molecular weight. Expression of constitutively active RhoA and cdc42 was confirmed using a Rho-GTPase activity assay comparing the constitutively active mutants to their isogenic partner lines.

### Transfection and Microscopy

#### Live-Cell Imaging

For colocalization and membrane analysis experiments, HeLa cells (LKB1-null) were co-transfected with either pEGFP-C1 control or wildtype LKB1 and its various constructs (Fig. 1a), Flag-STRADα, and LifeAct-mRuby (Ibidi, Madison, WI) to visualize f-actin filaments using Lipofectamine 2000 (Invitrogen), per manufacturer’s protocol. For paxillin experiments, cells were co-transfected with either pEGFP-C1 control or pEGFP-C1 LKB1 and various constructs, Flag-STRADα, and pmRuby-Paxillin-22. mRuby-Paxillin-22 was a gift from Michael Davidson (Addgene plasmid # 55877). 24 hours later, cells were imaged using a Perkin Elmer Ultraview spinning disk confocal microscope at 63x (1.40 NA) mounted onto a Zeiss Axiovert encased at 37 °C with 5% CO_2_. For colocalization and membrane analysis experiments, fluorescent images were acquired using a 488 and 568 nm laser line every 5 seconds for up to 5 minutes using a Hamamatsu Orca Flash 4.0 v2 CMOS camera with exposure times ranging from 250–1000 ms. For paxillin experiments, fluorescent images were acquired using a 488 and 568 nm laser line every 30 seconds for up to 30 minutes using a Hamamatsu Orca Flash 4.0 v2 CMOS camera with exposure times ranging from 250–1000 ms. For scratch wound assays, HeLa cells stably expressing our panel of GFP-LKB1 constructs were plated on a fibronectin-coated plate (40 ug/ml). 24 hours later, a confluent monolayer was scratched using a p10 tip and washed 3 times with PBS. Complete media was replaced, and cells were imaged using a Perkin Elmer Ultraview spinning disk confocal microscope at 10x (0.3 NA) mounted onto a Zeiss Axiovert encased at 37 °C with 5% CO_2_, with images acquired every 8 minutes for 21 hours.

#### Fixed Tissue Microscopy

HeLa cells (LKB1-null) were co-transfected with either pEGFP-C1 control or our panel of LKB1 constructs and Flag-STRADα using Lipofectamine 2000 (Invitrogen), per manufacturer’s protocol. 24 hours later, cells were fixed using Phemo buffer with a final concentration of: 3.7% paraformaldehyde (Electron Microscopy Sciences, Hatfield, PA), 0.05% glutaraldehyde (MP Biomedicals, Santa Ana, CA), and 0.5% Triton-X (Promega, Madison, WI) for 10 minutes at room temperature. Fixed cells were rinsed with PBS and stained with Alexa Fluor^®^ 555 Phalloidin (1:200 in PBS) for 1 hour, rinsed three times with PBS, and stained with 350 nM DAPI for 10 minutes followed by three more PBS washes. Coverslips were then mounted with ProLong^®^ Diamond Antifade Mountant. Fixed cells were imaged using a Leica SP8 inverted confocal microscope at 63x (HP PL APO 1.40 NA oil) using a 488 and 514 nm argon laser.

### Drug Treatments

#### ROCK Inhibitor

Cells were plated and transfected as previously described. 1 hour prior to fixation, the ROCK inhibitor Y-27632 (Millipore) was added at 10 μM to fresh complete DMEM. After 1 hour, cells were fixed as described.

#### FAK Inhibitor

Cells were plated and transfected as previously described. 8 hours after transfection, media was replaced with complete DMEM containing 1 μM FAK inhibitor PF-562271 (Pfizer) and incubated overnight. The next day, media was replaced with fresh DMEM with 1 μM PF-562271, and 1 hour later cells were imaged as described.

### Image Analysis

#### Stress Fiber Quantification

Images were analyzed using ImageJ/Fiji (NIH, Bethesda, MD) image analysis software. Stress fiber formation was quantified through manual counting of cells containing lateral stress fibers. A stress fiber was defined as an actin filament across the lateral width of the cell: If the cell expressed one or more stress fibers, we mark it as positive, and if not we define it as negative (Supplementary Figure 1 gives examples of each). For Fig. 1c, each HeLa GFP-LKB1 cell line was compared with the respective empty GFP control lines and also to its farnesylation mutant partner (WT vs. C430S, K78I vs. K78I-C430S, CTD vs. CTD-C430S) using a 2-tailed Chi-squared analysis with a p-value of 0.05. ****p ≤ 0.0001. All analysis was conducted over 3 separate experiments. In Fig. 1, Empty GFP: n = 103 cells; LKB1 WT: n = 150 cells; LKB1 C430S: n = 92 cells; LKB1 K78I: n = 198 cells; LKB1 K78I-C430S: n = 121 cells; LKB1 CTD: n = 142 cells; LKB1 CTD-C430S: n = 145 cells. Additionally (Supplementary Figure 1), the percent of stress fibers per field was quantified. These data passed the assumptions of parametric tests (homogeneity of variance), and this percentage was then analyzed using an ANOVA test, which showed statistical significance at a p-value of 0.05. Sidak’s multiple comparisons test was then performed to compare each construct to empty GFP control and its farnesylation mutant partner with a p-value of 0.05. ****p ≤ 0.0001. n = 3 fields of view/construct. In Fig. 2, Part B was broken into parts i and ii for simplicity, and each construct was compared to Empty GFP control, as well as within each family (ie: Empty GFP vs Empty GFP + RhoA Q63L and Empty GFP + cdc42 Q61L), using a 2-tailed Chi-squared analysis with a p-value of 0.05. In Fig. 2d, each construct was compared to empty GFP control as well as the respective treated vs untreated control (ie: empty GFP vs empty GFP + ROCKin) using a 2-tailed Chi-squared analysis with a p-value of 0.05. ****p ≤ 0.0001. In Fig. 2b(i), Empty GFP: n = 29 cells; Empty GFP + RhoA Q63L: n = 62 cells; Empty GFP + cdc42 Q61L: n = 40 cells; LKB1 Wildtype: n = 40 cells; LKB1 Wildtype + RhoA Q63L: n = 60 cells; LKB1 Wildtype + cdc42 Q61L: n = 82 cells; LKB1 C430S: n = 40 cells; LKB1 C430S + RhoA Q63L: n = 88 cells; LKB1 C430S + cdc42 Q61L: 103 cells. In Fig. 2b(ii), the Empty GFP, Empty GFP + RhoA Q63L, and Empty GFP + cdc42 Q61L values were repeated from Fig. 2b(i), LKB1 CTD: n = 36 cells; LKB1 CTD + RhoA Q63L: n = 86 cells; LKB1 CTD + cdc42 Q61L: 76 cells; LKB1 CTD-C430S: n = 45 cells; LKB1 CTD-C430S + RhoA Q63L: n = 52 cells; LKB1 CTD-C430S + cdc42 Q61L: n = 62 cells. In Fig. 2D, Empty GFP: n = 103 cells; Empty GFP + Y-27632: n = 110 cells; LKB1 Wildtype: n = 110 cells; LKB1 Wildtype + Y-27632: n = 92 cells; LKB1 CTD: n = 128 cells; LKB1 CTD + Y-27632: n = 101 cells.

#### Colocalization and Membrane Dynamics

In order to study dynamic protein colocalization and the relationships between membrane dynamics and colocalization we developed several enhancements to Machacek and Danuser’s CellEdgeTracker algorithm^35^,^45^. CellEdgeTracker software provides boundary tracking and discretization. We used CellEdgeTracker as a platform to develop Cellular Analysis of Dynamic Events (CADE) which uses tracked discretized boundaries to measure protein colocalization dynamics and associations between colocalization membrane motion dynamics. Time-lapse fluorescent images were first segmented using 3D graph cuts algorithm developed by ref. 46 and analyzed with CellEdgeTracker to obtain membrane positions {(*x(t*), *y(t*), *t*)}. The boundary was then divided into a sequence of sectors approximately 10 μm in length. The velocity *V* of each sector is calculated by equation (1), where d is the displacement vector and n is the normal vector for sector *i*. Normalized *V* is plotted in a heat map as a function of time (x-axis) and the position around the cell edge (left y-axis). Heatmap color is used to encode boundary velocity with red, blue and green denoting protrusion (*V* = 1), retraction (*V* = −1) and quiescence (*V* = 0) of the cell edge velocity respectively.


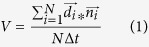


Based on the cell membrane algorithm, we developed our colocalization algorithm to quantify the spatiotemporal colocalization of two proteins. We first erode the binary cell mask to obtain a 2 μm * 10 μm band at the cell edge, and then partition this band into *N* contiguous regions where protein expression *G, R* and colocalization signals between green and red channels c*oloc(G,R*) can be quantified as in equations (2),(3),(4), where and denote the average background signals from green and red channels, respectively. *μ*_*G*_ and *μ*_*R*_ are the mean value of green and red signals. *Cov* and *Var* are the covariance and the variance of the mean subtracted green and red signals, respectively. The normalized colocalization is finally plotted as a heat map both in time and spatial domain. Red indicates high colocalization (*coloc* = 1) and blue indicates low colocalization (*coloc* = 0).


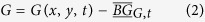



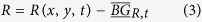



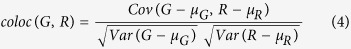


To investigate the spatiotemporal relationship *C* between membrane dynamics and protein colocalization, we calculated the dot product between *V* and *coloc* using equation (5), where *N* and *t* are the number of rows and columns in velocity heat maps. *C* is also normalized according to the maximum and minimum *C* values among all cells. Similarly as velocity and colocalization heat maps, normalized *C* is plotted into heat maps. Red indicates highly coupled protrusion and high colocalization (*C* = 1); Orange indicates highly coupled protrusion and low colocalization (*C* = 0.5); Green indicates coupling between quiescence and low colocalization (*C* = 0); Cyan indicates coupling between retraction and low colocalization (*C* = −0.5) and blue indicates highly coupled retraction and high colocalization (*C* = −1).


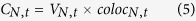


In Fig. 3, significance was initially measured using a Kruskal-Wallis test with a p-value of 0.05, which showed statistical significance. A Dunn’s multiple comparisons test was then performed to compare each construct to empty GFP control and its farnesylation mutant partner with a p-value of 0.05, where **p ≤ 0.01; ***p ≤ 0.001; ****p ≤ 0.0001. In Fig. 3b, Empty GFP: n = 16 cells; LKB1 Wildtype: n = 17 cells; LKB1 C430S: n = 16 cells; LKB1 K78I: n = 15 cells; LKB1 K78I-C430S: n = 14 cells; LKB1 CTD: n = 14 cells; LKB1 CTD-C430S: n = 15 cells. In Fig. 3e–f, LKB1 Wildtype: n = 9 cells; LKB1 K78I: n = 7 cells; LKB1 CTD: n = 5 cells.

#### Quantification of Membrane Dynamic Patterns

We developed a semi-automated graphical user interface to facilitate measuring protrusion/retraction regions on the membrane velocity heatmap. Several regions of interest (ROIs) were manually selected on the heatmap to represent the protrusion/retraction regions using CROIEditor algorithm written by Jonas Reber (http://www.mathworks.com/matlabcentral/fileexchange/31388-multi-roi-mask-editor-class/content/CROIEditor/CROIEditor.m). Then these ROIs were segmented into binary masks. Several morphology features for each ROI was calculated using regionprops function in Matlab. The features for each ROI are the number of ROIs, the area of the ROI, angle of the ROI from major axis to the x-axis, and the duration time, which is the length in x-axis converted to time unit. Since there is no standard definition of a protrusion/retraction region, we asked multiple users to select the ROIs for the same heatmap, similar to refs 35,45,47 and 48. We then performed a one-way ANOVA test amongst all users to check reproducibility and found the p-value larger than 0.05, which indicated the selection among different users was similar. The ROIs of the multiple users was then averaged for statistical analysis. Colocalization duration and events/cell/minute (Fig. 3e–f) was compared between empty GFP control, LKB1 K78I, and LKB1 CTD using a Kruskal-Wallis test with a p-value of 0.05, which showed statistical significance. A Dunn’s multiple comparison test was then performed to compare each construct to each other with a p-value of 0.05. In Figs 4 and 5, membrane event duration, angle, and number of events/cell/minute was measured using a Kruskal-Wallis test with a p-value of 0.05, which showed statistical significance. A Dunn’s multiple comparisons test was then performed to compare each construct to empty GFP control and its farnesylation mutant partner with a p-value of 0.05. In Figs 4f and 5d, a 2-tailed Chi-squared analysis was performed with a p-value of 0.05. *p ≤ 0.05; **p ≤ 0.01, ***p ≤ 0.001, ****p ≤ 0.0001. Values were determined over three separate experiments. In Fig. 4, Empty GFP: n = 7 cells; LKB1 Wildtype: n = 9 cells; LKB1 C430S: n = 8 cells; LKB1 K78I: n = 8 cells; LKB1 CTD: n = 5 cells; LKB1 CTD-C430S: n = 7 cells. In Fig. 5, Empty GFP: n = 7 cells; Empty GFP + FAKin: n = 5 cells; LKB1 K78I: n = 8 cells; LKB1 K78I + FAKin: n = 5 cells; LKB1 CTD: n = 5 cells; LKB1 CTD + FAKin: n = 5 cells.

#### Paxillin Adhesion

Volocity (PerkinElmer) image analysis software was used to quantify fluorescent paxillin sites. A ROI was drawn around a single lamellipodia and the image was cropped around the ROI. The images were then thresholded and the smoothing filter was used. Automatic detection of objects was employed and objects less than a volume of 0.1 μm^3^ were excluded from the analysis. We manually identified the cell boundary for each image and used this for the analysis. Fluorescent site area, elongation, and distance from the membrane were then analyzed and recorded. The site area, elongation, and distance to membrane of each HeLa GFP-LKB1 cell line was measured using a Kruskal-Wallis test with a p-value of 0.05, which showed statistical significance. A Dunn’s multiple comparison test was then performed to compare each construct to empty GFP control and LKB1 wildtype with a p-value of 0.05, *p ≤ 0.05; **p ≤ 0.01, ***p ≤ 0.001, ****p ≤ 0.0001. N = 10 cells/condition over 3 separate experiments.

#### Scratch wound analysis

Volocity (Perkin Elmer, Waltham, MA) image analysis software and manual tracking was used to quantify meandering index and percent area invaded. An ROI was manually drawn around the scratch wound at 0, 3, 6, 9, 12, 15, 18, and 21 hours, and the percent area closed was quantified over time. Meandering index (displacement/distance) and area closed was measured using a Kruskal-Wallis test with a p-value of 0.05, which showed statistical significance. A Dunn’s multiple comparisons test was then performed to compare each construct to empty GFP control and LKB1 wildtype with a p-value of 0.05. Wound closure passed the assumptions of parametric tests (homogeneity of variance) and was thus measured using ANOVA, which showed statistical significance. We then performed a Sidak’s multiple comparisons test to compare each construct to empty GFP control and LKB1 wildtype at 3 and 6 hours with a p-value of 0.05. * ≤ 0.05; **≤0.01; ***≤0.001; ****≤0.0001. n = 30 cells, 3 scratches/condition, over 3 separate experiments.

#### Subcellular localization

Non-confluent cells were imaged, and image analysis using Imaris Cell (Bitplane, South Windsor, CT) was performed using the cell tracking function. Cytoplasmic LKB1 was identified using phalloidin as a cytoplasmic marker, while nuclear LKB1 was identified using DAPI. Mean intensity of LKB1 was quantified in both the cytoplasmic and nuclear regions, and the cytoplasmic:nuclear ratio was determined for each cell. C:N ratio was compared between wildtype LKB1 and the various constructs, as well as the isogenic parental and farnesyl-mutant condition (WT vs C430S, K78I vs K78I-C430S, CTD vs CTD-C430S), using the Dunn’s multiple comparisons test with a p-value of 0.05. *≤0.05; ***≤0.001; ****≤0.0001. Empty GFP: n = 15 cells; LKB1 Wildtype: n = 21 cells; LKB1 C430S: n = 20 cells; LKB1 K78I: n = 17 cells; LKB1 K78I-C430S: n = 16 cells; LKB1 CTD: n = 21 cells; LKB1 CTD-C430S: n = 20 cells.

## Additional Information

**How to cite this article**: Wilkinson, S. *et al*. Coordinated cell motility is regulated by a combination of LKB1 farnesylation and kinase activity. *Sci. Rep.* **7**, 40929; doi: 10.1038/srep40929 (2017).

**Publisher's note:** Springer Nature remains neutral with regard to jurisdictional claims in published maps and institutional affiliations.

## Supplementary Material

Supplementary Information

## Figures and Tables

**Table 1:** Differential effects of LKB1 protein domains on cell motility machinery.

LKB1 Protein Domain Functions		
	LKB1 Kinase Activity	LKB1 Farnesylation
Actin stress fiber assembly	+	++
Actin colocalization	−	+
Membrane Dynamics/Traveling Waves	+	−
Nascent Adhesion Area/Length	+	+
Nascent Adhesion Localization	−	+

LKB1 kinase activity impacts HeLa cell stress fiber assembly, represses traveling waves during membrane activity, and promotes nascent adhesion (NA) area and elongation. Conversely, LKB1 farnesylation is critical for stress fiber assembly, its actin colocalization, NA area and elongation, and NA localization to the leading edge membrane.

## References

[b1] Hawley S. A. . Complexes between the LKB1 tumor suppressor, STRAD alpha/beta and MO25 alpha/beta are upstream kinases in the AMP-activated protein kinase cascade. Journal of biology 2, 28, doi: 10.1186/1475-4924-2-28 (2003). 14511394 PMC333410

[b2] Shaw R. J. . The tumor suppressor LKB1 kinase directly activates AMP-activated kinase and regulates apoptosis in response to energy stress. Proceedings of the National Academy of Sciences of the United States of America 101, 3329–3335, doi: 10.1073/pnas.0308061100 (2004). 14985505 PMC373461

[b3] Asada N., Sanada K. & Fukada Y. LKB1 regulates neuronal migration and neuronal differentiation in the developing neocortex through centrosomal positioning. The Journal of neuroscience: the official journal of the Society for Neuroscience 27, 11769–11775, doi: 10.1523/JNEUROSCI.1938-07.2007 (2007). 17959818 PMC6673218

[b4] Etienne-Manneville S. Cdc42–the centre of polarity. J Cell Sci 117, 1291–1300, doi: 10.1242/jcs.01115 (2004). 15020669

[b5] Konen J. . LKB1 kinase-dependent and -independent defects disrupt polarity and adhesion signaling to drive collagen remodeling during invasion. Molecular biology of the cell 27, 1069–1084, doi: 10.1091/mbc.E15-08-0569 (2016). 26864623 PMC4814216

[b6] Marcus A. I. & Zhou W. LKB1 regulated pathways in lung cancer invasion and metastasis. Journal of thoracic oncology: official publication of the International Association for the Study of Lung Cancer 5, 1883–1886, doi: 10.1097/JTO.0b013e3181fbc28a (2010). PMC2995373 21102257

[b7] Nakano A. & Takashima S. LKB1 and AMP-activated protein kinase: regulators of cell polarity. Genes to cells: devoted to molecular & cellular mechanisms 17, 737–747, doi: 10.1111/j.1365-2443.2012.01629.x (2012). 22892070 PMC3533759

[b8] Chan K. T. . LKB1 loss in melanoma disrupts directional migration toward extracellular matrix cues. The Journal of cell biology 207, 299–315, doi: 10.1083/jcb.201404067 (2014). 25349262 PMC4210439

[b9] Goodwin J. M. . An AMPK-independent signaling pathway downstream of the LKB1 tumor suppressor controls Snail1 and metastatic potential. Molecular cell 55, 436–450, doi: 10.1016/j.molcel.2014.06.021 (2014). 25042806 PMC4151130

[b10] Kline E. R., Shupe J., Gilbert-Ross M., Zhou W. & Marcus A. I. LKB1 represses focal adhesion kinase (FAK) signaling via a FAK-LKB1 complex to regulate FAK site maturation and directional persistence. The Journal of biological chemistry 288, 17663–17674, doi: 10.1074/jbc.M112.444620 (2013). 23637231 PMC3682567

[b11] Barnes A. P. . LKB1 and SAD kinases define a pathway required for the polarization of cortical neurons. Cell 129, 549–563, doi: 10.1016/j.cell.2007.03.025 (2007). 17482548

[b12] Kishi M., Pan Y. A., Crump J. G. & Sanes J. R. Mammalian SAD kinases are required for neuronal polarization. Science 307, 929–932, doi: 10.1126/science.1107403 (2005). 15705853

[b13] Ding L. . Somatic mutations affect key pathways in lung adenocarcinoma. Nature 455, 1069–1075, doi: 10.1038/nature07423 (2008). 18948947 PMC2694412

[b14] Matsumoto S. . Prevalence and specificity of LKB1 genetic alterations in lung cancers. Oncogene 26, 5911–5918, doi: 10.1038/sj.onc.1210418 (2007). 17384680 PMC3457639

[b15] Sanchez-Cespedes M. . Inactivation of LKB1/STK11 is a common event in adenocarcinomas of the lung. Cancer Res 62, 3659–3662 (2002). 12097271

[b16] The Cancer Genome Atlas Research, N. Comprehensive molecular profiling of lung adenocarcinoma. Nature 511, 543–550, doi: 10.1038/nature13385 (2014). 25079552 PMC4231481

[b17] Wingo S. N. . Somatic LKB1 mutations promote cervical cancer progression. PLoS One 4, e5137, doi: 10.1371/journal.pone.0005137 (2009). 19340305 PMC2660434

[b18] Ji H. . LKB1 modulates lung cancer differentiation and metastasis. Nature 448, 807–810, doi: 10.1038/nature06030 (2007). 17676035

[b19] Hezel A. F. & Bardeesy N. LKB1; linking cell structure and tumor suppression. Oncogene 27, 6908–6919, doi: 10.1038/onc.2008.342 (2008). 19029933

[b20] Houde V. P. . Investigation of LKB1 Ser431 phosphorylation and Cys433 farnesylation using mouse knockin analysis reveals an unexpected role of prenylation in regulating AMPK activity. The Biochemical journal 458, 41–56, doi: 10.1042/BJ20131324 (2014). 24295069 PMC3898322

[b21] Collins S. P., Reoma J. L., Gamm D. M. & Uhler M. D. LKB1, a novel serine/threonine protein kinase and potential tumour suppressor, is phosphorylated by cAMP-dependent protein kinase (PKA) and prenylated *in vivo*. The Biochemical journal 345 Pt 3, 673–680 (2000). 10642527 PMC1220803

[b22] Sapkota G. P. . Phosphorylation of the protein kinase mutated in Peutz-Jeghers cancer syndrome, LKB1/STK11, at Ser431 by p90(RSK) and cAMP-dependent protein kinase, but not its farnesylation at Cys(433), is essential for LKB1 to suppress cell vrowth. The Journal of biological chemistry 276, 19469–19482, doi: 10.1074/jbc.M009953200 (2001). 11297520

[b23] Baas A. F. . Complete polarization of single intestinal epithelial cells upon activation of LKB1 by STRAD. Cell 116, 457–466 (2004). 15016379 10.1016/s0092-8674(04)00114-x

[b24] Zhang S. . The tumor suppressor LKB1 regulates lung cancer cell polarity by mediating cdc42 recruitment and activity. Cancer Res 68, 740–748, doi: 10.1158/0008-5472.CAN-07-2989 (2008). 18245474

[b25] Xu X., Omelchenko T. & Hall A. LKB1 tumor suppressor protein regulates actin filament assembly through Rho and its exchange factor Dbl independently of kinase activity. BMC cell biology 11, 77, doi: 10.1186/1471-2121-11-77 (2010). 20939895 PMC2964536

[b26] Xu X., Jin D., Durgan J. & Hall A. LKB1 controls human bronchial epithelial morphogenesis through p114RhoGEF-dependent RhoA activation. Molecular and cellular biology 33, 2671–2682, doi: 10.1128/MCB.00154-13 (2013). 23648482 PMC3700127

[b27] Carretero J. . Integrative genomic and proteomic analyses identify targets for Lkb1-deficient metastatic lung tumors. Cancer cell 17, 547–559, doi: 10.1016/j.ccr.2010.04.026 (2010). 20541700 PMC2901842

[b28] Swaminathan V., Fischer R. S. & Waterman C. M. The FAK-Arp2/3 interaction promotes leading edge advance and haptosensing by coupling nascent adhesions to lamellipodia actin. Molecular biology of the cell 27, 1085–1100, doi: 10.1091/mbc.E15-08-0590 (2016). 26842895 PMC4814217

[b29] Mehenni H. . Loss of LKB1 kinase activity in Peutz-Jeghers syndrome, and evidence for allelic and locus heterogeneity. American journal of human genetics 63, 1641–1650 (1998). 9837816 10.1086/302159 PMC1377635

[b30] Katoh K. . Rho-kinase–mediated contraction of isolated stress fibers. The Journal of cell biology 153, 569–584 (2001). 11331307 10.1083/jcb.153.3.569 PMC2190572

[b31] Totsukawa G. . Distinct roles of MLCK and ROCK in the regulation of membrane protrusions and focal adhesion dynamics during cell migration of fibroblasts. The Journal of cell biology 164, 427–439, doi: 10.1083/jcb.200306172 (2004). 14757754 PMC2172229

[b32] Krause M. & Gautreau A. Steering cell migration: lamellipodium dynamics and the regulation of directional persistence. Nat Rev Mol Cell Biol 15, 577–590, doi: 10.1038/nrm3861 (2014). 25145849

[b33] Driscoll M. K., Losert W., Jacobson K. & Kapustina M. Spatiotemporal relationships between the cell shape and the actomyosin cortex of periodically protruding cells. Cytoskeleton (Hoboken, N.J.) 72, 268–281, doi: 10.1002/cm.21229 (2015). PMC4529805 26147497

[b34] Gerisch G. . Mobile actin clusters and traveling waves in cells recovering from actin depolymerization. Biophys J 87, 3493–3503, doi: 10.1529/biophysj.104.047589 (2004). 15347592 PMC1304815

[b35] Machacek M. & Danuser G. Morphodynamic profiling of protrusion phenotypes. Biophys J 90, 1439–1452, doi: 10.1529/biophysj.105.070383 (2006). 16326902 PMC1367294

[b36] Buccione R., Orth J. D. & McNiven M. A. Foot and mouth: podosomes, invadopodia and circular dorsal ruffles. Nat Rev Mol Cell Biol 5, 647–657, doi: 10.1038/nrm1436 (2004). 15366708

[b37] Stokes J. B. . Inhibition of focal adhesion kinase by PF-562, 271 inhibits the growth and metastasis of pancreatic cancer concomitant with altering the tumor microenvironment. Molecular cancer therapeutics 10, 2135–2145, doi: 10.1158/1535-7163.MCT-11-0261 (2011). 21903606 PMC3213273

[b38] Jay D. G. The clutch hypothesis revisited: ascribing the roles of actin-associated proteins in filopodial protrusion in the nerve growth cone. Journal of neurobiology 44, 114–125 (2000). 10934316 10.1002/1097-4695(200008)44:2<114::aid-neu3>3.0.co;2-8

[b39] Nabi I. R. The polarization of the motile cell. J Cell Sci 112 (Pt 12), 1803–1811 (1999). 10341200 10.1242/jcs.112.12.1803

[b40] Ishizaki T. . Pharmacological properties of Y-27632, a specific inhibitor of rho-associated kinases. Molecular pharmacology 57, 976–983 (2000). 10779382

[b41] Gao Y. . LKB1 inhibits lung cancer progression through lysyl oxidase and extracellular matrix remodeling. Proceedings of the National Academy of Sciences of the United States of America 107, 18892–18897, doi: 10.1073/pnas.1004952107 (2010). 20956321 PMC2973865

[b42] Carragher N. O. & Frame M. C. Focal adhesion and actin dynamics: a place where kinases and proteases meet to promote invasion. Trends Cell Biol 14, 241–249, doi: 10.1016/j.tcb.2004.03.011 (2004). 15130580

[b43] Kallergi G., Agelaki S., Markomanolaki H., Georgoulias V. & Stournaras C. Activation of FAK/PI3K/Rac1 signaling controls actin reorganization and inhibits cell motility in human cancer cells. Cellular physiology and biochemistry: international journal of experimental cellular physiology, biochemistry, and pharmacology 20, 977–986, doi: 10.1159/000110458 (2007). 17982280

[b44] Serrels B. . Focal adhesion kinase controls actin assembly via a FERM-mediated interaction with the Arp2/3 complex. Nature cell biology 9, 1046–1056, doi: 10.1038/ncb1626 (2007). 17721515

[b45] Machacek M. . Coordination of Rho GTPase activities during cell protrusion. Nature 461, 99–103, doi: 10.1038/nature08242 (2009). 19693013 PMC2885353

[b46] Yuan J., Bae E. & Tai X.-C. In 2010 IEEE Conference on Computer Vision and Pattern Recognition (CVPR). 2217–2224 (IEEE).

[b47] Ryan G. L., Petroccia H. M., Watanabe N. & Vavylonis D. Excitable actin dynamics in lamellipodial protrusion and retraction. Biophys J 102, 1493–1502, doi: 10.1016/j.bpj.2012.03.005 (2012). 22500749 PMC3318140

[b48] Zhuo Y. . Subcellular and Dynamic Coordination between Src Activity and Cell Protrusion in Microenvironment. Scientific reports 5, 12963, doi: 10.1038/srep12963 (2015). 26261043 PMC4531316

