# Supplementary material for: Coordinated cell motility is regulated by a combination of LKB1 farnesylation and kinase activity
Source: Sci Rep. 2017 Jan 19;7:40929. doi: 10.1038/srep40929 (PMC5244416; doi:10.1038/srep40929)
Supplement: Supplementary Information [file srep40929-s1.pdf]

Coordinated cell motility is regulated by a combination of LKB1 farnesylation and kinase activity

S Wilkinson[1, 2], Y Hou[1, 3], JT Zoine[1, 2], J Saltz[4], C Zhang[5], Z Chen[5], LAD Cooper[1,3], AI Marcus[1*]

Affiliations:

[1] Winship Cancer Institute of Emory University, Department of Hematology and Medical Oncology

[2] Graduate Program in Cancer Biology, Emory University

[3] Department of Biomedical Informatics, Emory University

[4] Department of Biomedical Informatics, Stony Brook University

[5] Department of Biostatistics and Bioinformatics, Emory University

* Corresponding Author: Dr. Adam Marcus, 1365 Clifton Road, Building C, Room 4092, Atlanta, GA 30322. Phone: 404-778-4597; Fax: 404-778-5530. aimarcu@emory.edu.

Supplementary Information

Supplementary Figure Legends

Supplementary Figure 1: LKB1 farnesylation drives actin stress fiber assembly. (A) Further examples of defining a cell as containing stress fibers. Top row shows examples of cells containing at least one stress fiber spanning the lateral width of the cell. These cells are marked positive. Example stress fibers indicated by red arrows. Bottom row shows examples of cells lacking stress fibers spanning the lateral width of the cell; these cells are marked negative. (B-D) Percent cells exhibiting lateral stress fibers per field. (B) Images from Figure 1A were analyzed to determine the percent stress fiber-positive cells per field. (C) Images from Figure 2D were analyzed to determine the percent stress fiber-positive cells per field. (Di, ii) Images from Figure 2Bi, ii were analyzed to determine the percent stress fiber-positive cells per field. For B-D, data passed assumptions of parametric tests (homogeneity of variance), and was then analyzed using ANOVA with a p-value of 0.05. Upon finding significance, we compared each construct to empty GFP control and its respective farnesyl-mutant

partner (WT vs C430S, etc) using Sidak's multiple comparisons test with a p-value of 0.05. ****=p≤0.0001. Error bars = SEM

Supplementary Figure 2: LKB1 farnesylation promotes its cytoplasmic localization. (A) Our panel of GFP-LKB1 constructs was transfected into HeLa (LKB1-null) cells. 24 hours later, cells were fixed and stained with phalloidin and DAPI. (B) Mean cytoplasmic and nuclear intensities GFP-LKB1 were calculated in each cell to calculate a Cytoplasmic:Nuclear (C:N) ratio. A C:N ratio of 1:1 indicates an even amount of LKB1 in the cytoplasm and nucleus, while a C:N of 0.5 indicates twice as much nuclear LKB1 as cytoplasmic. (C) Average cell footprint was measured in non-confluent cells. Significance was initially measured using a Kruskal-Wallis test with a p-value of 0.05, which showed statistical significances. It was then measured between comparisons using a Dunn's multiple comparisons test, where *=p≤0.05; ***=p≤0.001; ****=p≤0.0001. Scale bar: 10 µm. Error bars = SEM.

Supplementary Figure 3: Further examples of persistence of LKB1-actin colocalization. (A) 2 examples, with colocalization events identified, of CADE analysis of HeLa cells re-expressing LKB1 wildtype. (B) 2 examples, with colocalization events identified, of CADE analysis of HeLa cells re-expressing LKB1 C-terminal domain. (C) 2 examples, with colocalization events identified, of CADE analysis of HeLa cells re-expressing LKB1 K78I.

Supplementary Figure 4: Further examples of cellular membrane dynamics. (A) Membrane analysis, with ROIs identified, of HeLa cells re-expressing empty GFP control. (B-F) Membrane analysis, with ROIs identified, of LKB1 wildtype and the various LKB1 constructs.

Supplementary Figure 5: Further examples of cellular membrane dynamics on FAK inhibition. (A) 2 examples, with ROIs identified, of CADE analysis of HeLa cells re-expressing empty GFP control with 1 µM PF-562271 FAK inhibitor. (B) 2 examples, with ROIs identified, of CADE analysis of HeLa cells re-expressing LKB1 K78I with 1 µM PF-562271 FAK inhibitor. (C) 2 examples, with ROIs identified, of CADE analysis of HeLa cells re-expressing LKB1 CTD with 1 µM PF-562271 FAK inhibitor.

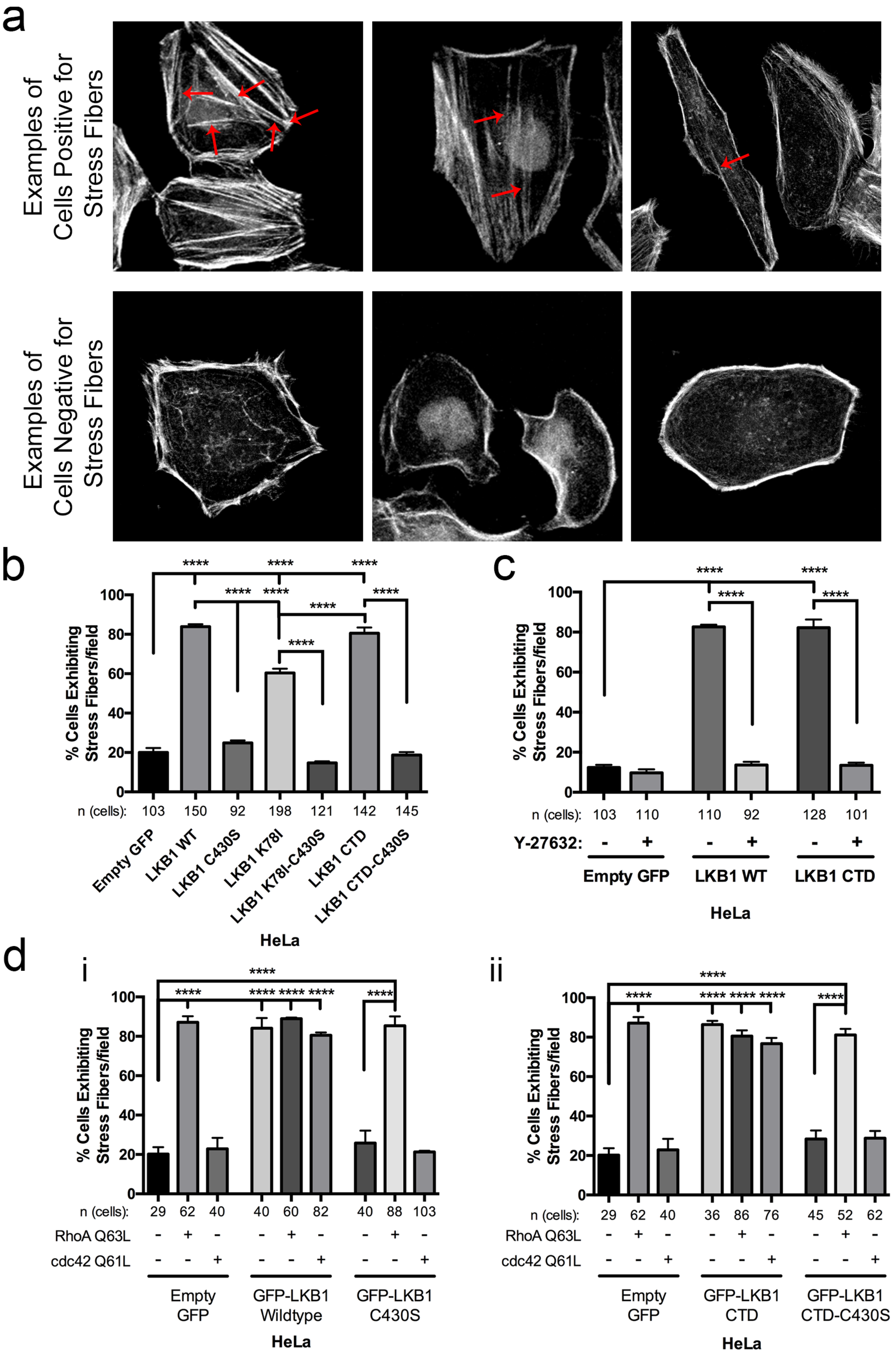
a
Examples of Cells Positive for Stress Fibers
Examples of Cells Negative for Stress Fibers
b
% Cells Exhibiting Stress Fibers/field
****
n (cells): 103 150 92 198 121 142 145
Empty GFP
LKB1 WT
LKB1 C430S
LKB1 K78I
LKB1 K78I-C430S
LKB1 CTD
LKB1 CTD-C430S
HeLa
c
% Cells Exhibiting Stress Fibers/field
****
n (cells): 103 110 110 92 128 101
Y-27632: - + - + - +
Empty GFP
LKB1 WT
LKB1 CTD
HeLa
d
i
% Cells Exhibiting Stress Fibers/field
****
n (cells): 29 62 40 40 60 82 40 88 103
RhoA Q63L - + - - + - - + -
cdc42 Q61L - - + - - + - - +
Empty GFP
GFP-LKB1 Wildtype
GFP-LKB1 C430S
HeLa
ii
% Cells Exhibiting Stress Fibers/field
****
n (cells): 29 62 40 36 86 76 45 52 62
RhoA Q63L - + - - + - - + -
cdc42 Q61L - - + - - + - - +
Empty GFP
GFP-LKB1 CTD
GFP-LKB1 CTD-C430S
HeLa

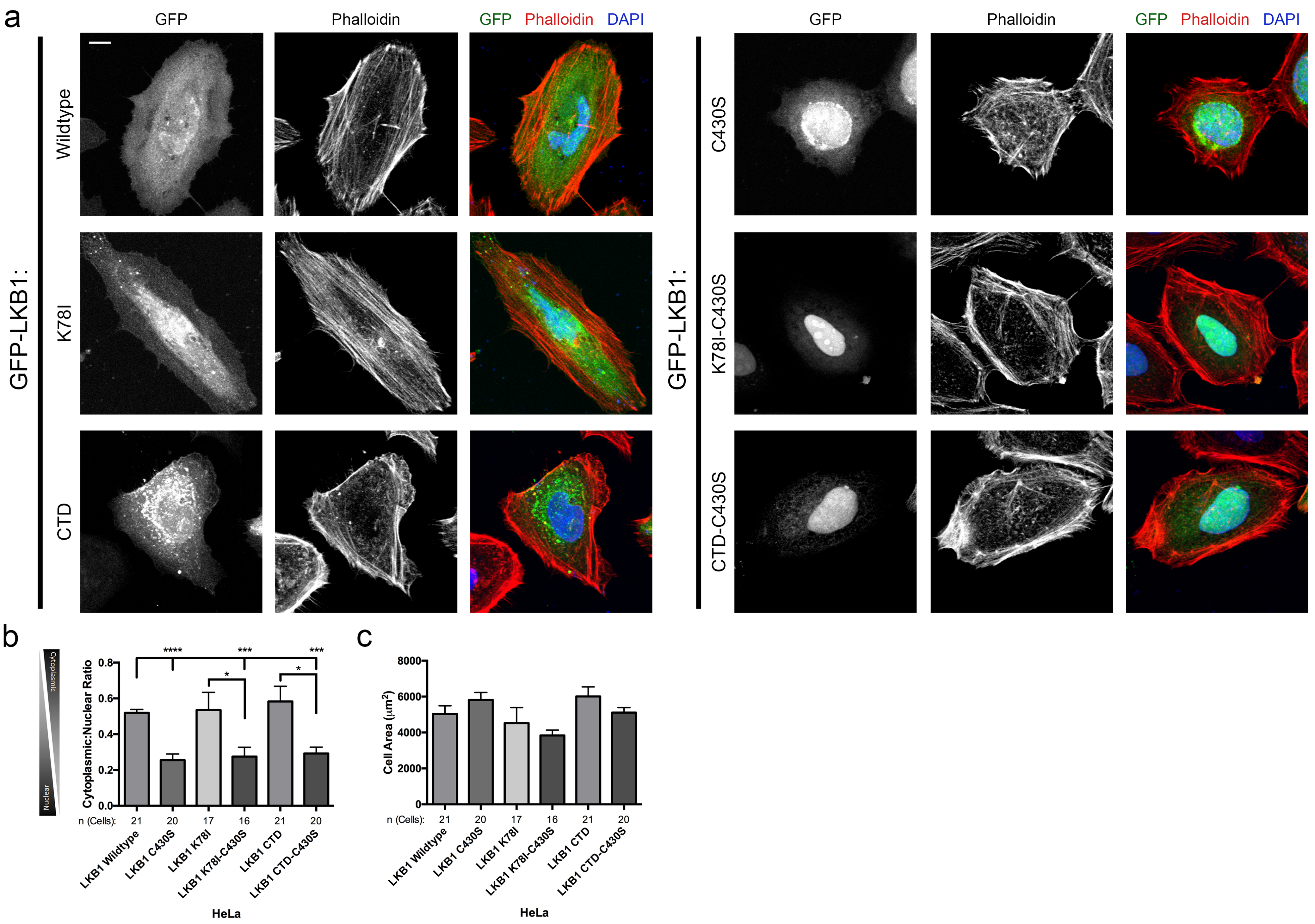
a
GFP
Phalloidin
GFP Phalloidin DAPI
GFP-LKB1:
Wildtype
K78I
CTD
C430S
K78I-C430S
CTD-C430S
b
Cytoplasmic
Nuclear
Cytoplasmic:Nuclear Ratio
0.0
0.2
0.4
0.6
0.8
****
***
***
*
*
n (Cells): 21 20 17 16 21 20
LKB1 Wildtype
LKB1 C430S
LKB1 K78I
LKB1 K78I-C430S
LKB1 CTD
LKB1 CTD-C430S
HeLa
c
Cell Area (μm²)
0
2000
4000
6000
8000
n (Cells): 21 20 17 16 21 20
LKB1 Wildtype
LKB1 C430S
LKB1 K78I
LKB1 K78I-C430S
LKB1 CTD
LKB1 CTD-C430S
HeLa

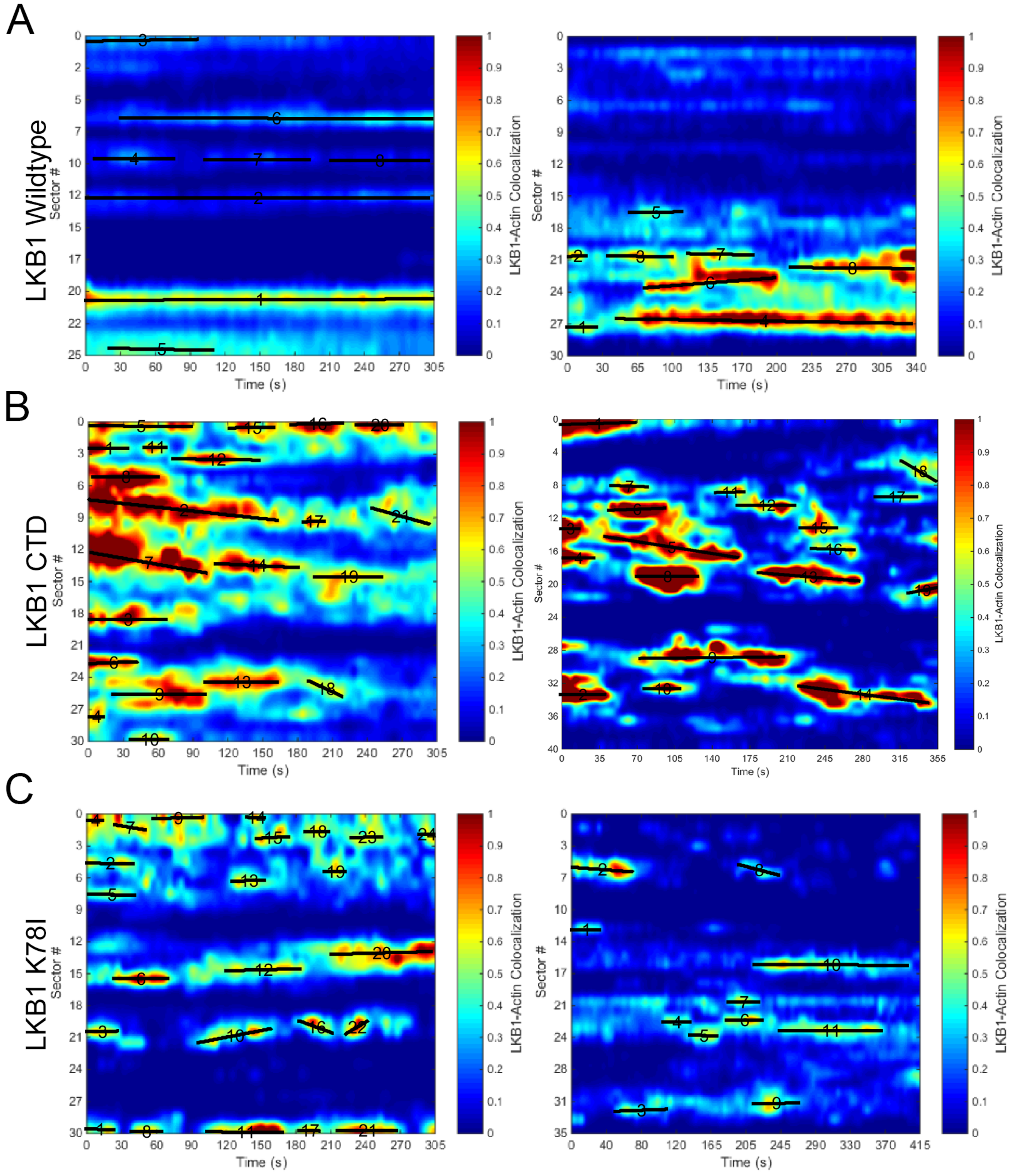

A
LKB1 Wildtype
B
LKB1 CTD
C
LKB1 K78I
Sector #
Time (s)
LKB1-Actin Colocalization

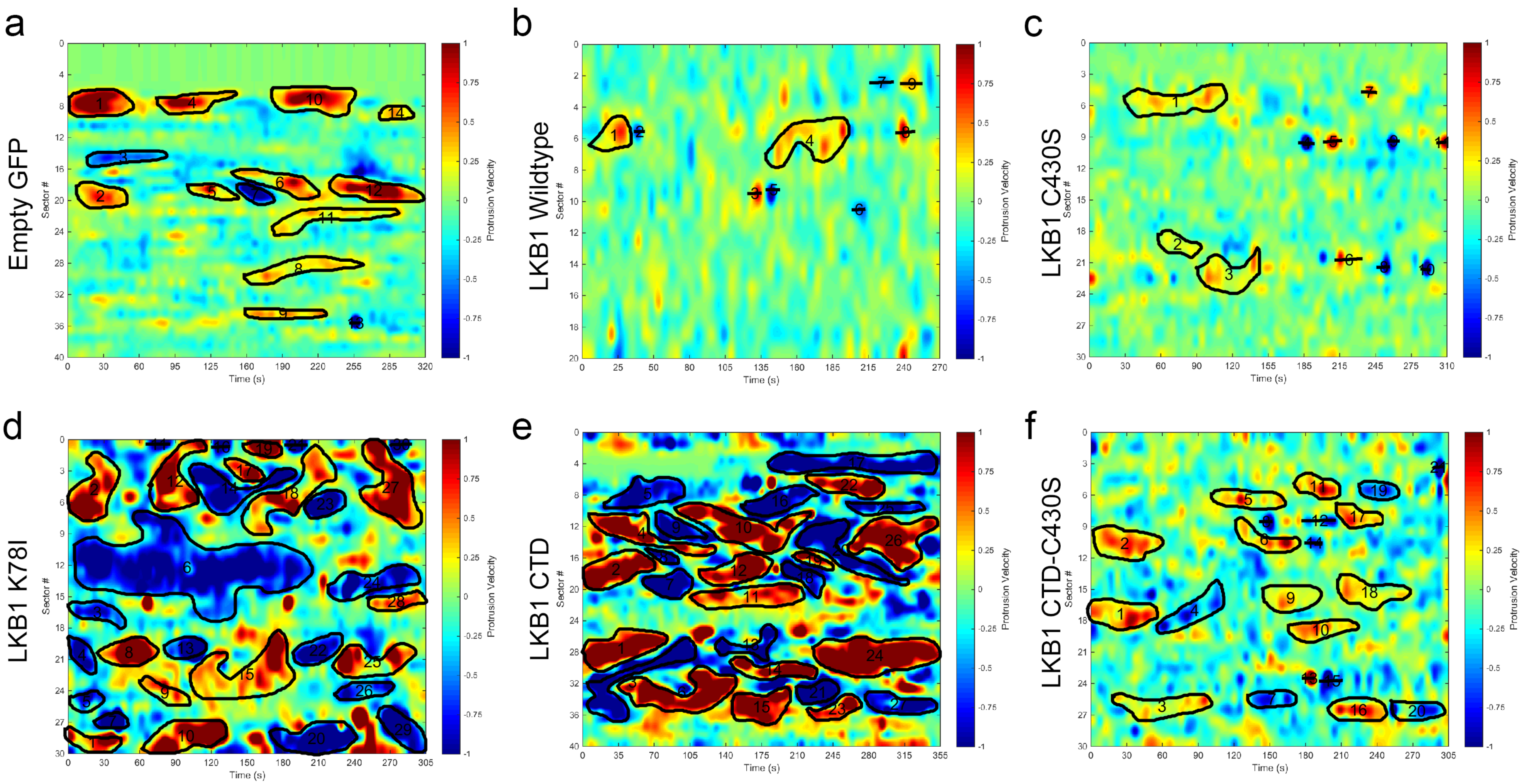

a
Empty GFP
b
LKB1 Wildtype
c
LKB1 C430S
d
LKB1 K78I
e
LKB1 CTD
f
LKB1 CTD-C430S
Sector #
Time (s)
Protrusion Velocity

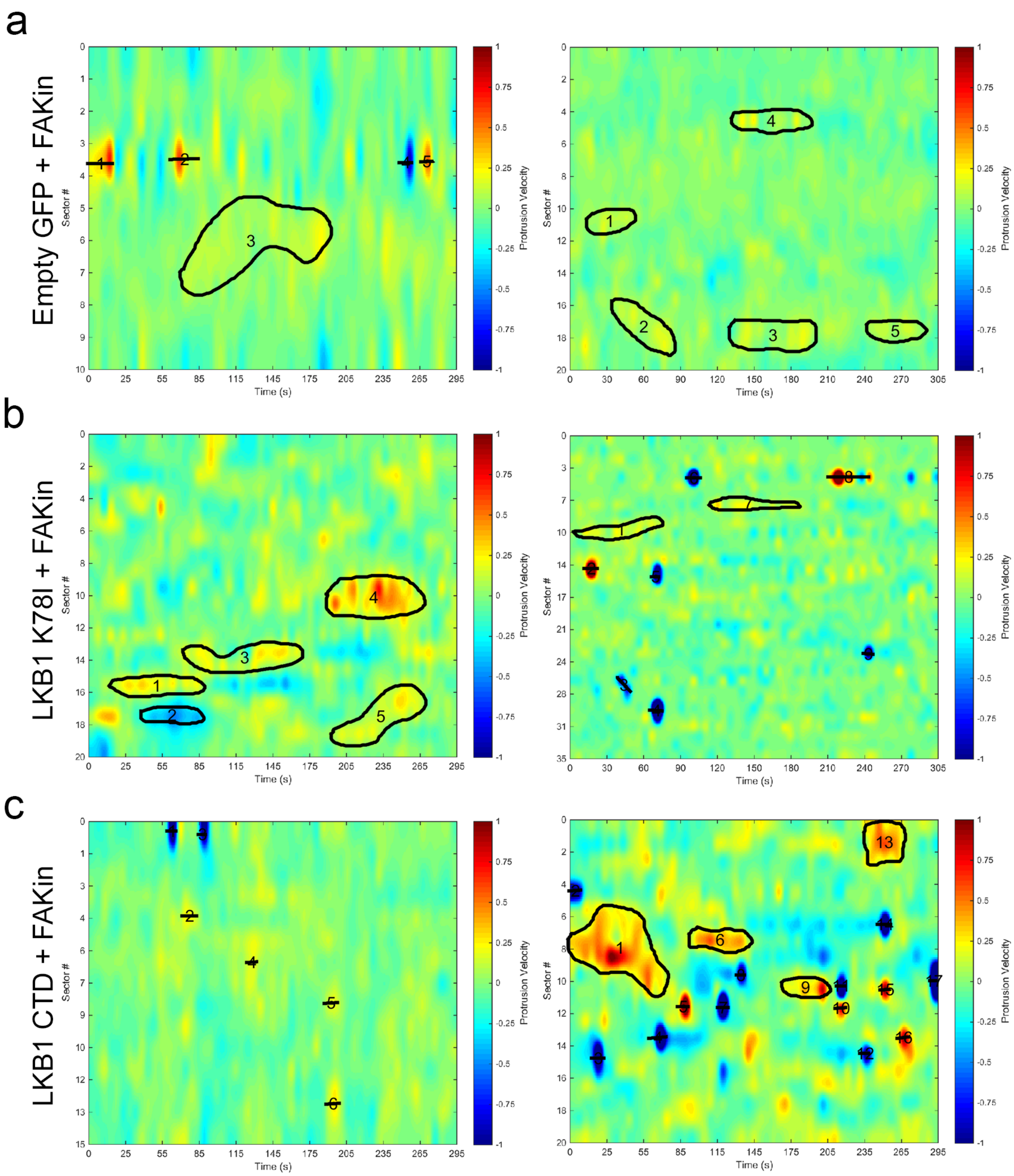

a
Empty GFP + FAKin
b
LKB1 K78I + FAKin
c
LKB1 CTD + FAKin
Sector #
Time (s)
Protrusion Velocity
